# Correction: Non-invasive detection of urothelial cancer through the analysis of driver gene mutations and aneuploidy

**DOI:** 10.7554/eLife.43237

**Published:** 2018-11-12

**Authors:** Simeon U Springer, Chung-Hsin Chen, Maria Del Carmen Rodriguez Pena, Lu Li, Christopher Douville, Yuxuan Wang, Joshua David Cohen, Diana Taheri, Natalie Silliman, Joy Schaefer, Janine Ptak, Lisa Dobbyn, Maria Papoli, Isaac Kinde, Bahman Afsari, Aline C Tregnago, Stephania M Bezerra, Christopher VandenBussche, Kazutoshi Fujita, Dilek Ertoy, Isabela W Cunha, Lijia Yu, Trinity J Bivalacqua, Arthur P Grollman, Luis A Diaz, Rachel Karchin, Ludmila Danilova, Chao-Yuan Huang, Chia-Tung Shun, Robert J Turesky, Byeong Hwa Yun, Thomas A Rosenquist, Yeong-Shiau Pu, Ralph H Hruban, Cristian Tomasetti, Nickolas Papadopoulos, Ken W Kinzler, Bert Vogelstein, Kathleen G Dickman, George J Netto

Springer SU, Chen C-H, Rodriguez Pena MDC, Li L, Douville C, Wang Y, Cohen JD, Taheri D, Silliman N, Schaefer J, Ptak J, Dobbyn L, Papoli M, Kinde I, Afsari B, Tregnago AC, Bezerra SM, VandenBussche C, Fujita K, Ertoy D, Cunha IW, Yu L, Bivalacqua TJ, Grollman AP, Diaz LA, Karchin R, Danilova L, Huang C-Y, Shun C-T, Turesky RJ, Yun BH, Rosenquist TA, Pu Y-S, Hruban RH, Tomasetti C, Papadopoulos N, Kinzler KW, Vogelstein B, Dickman KG, Netto GJ. 2018. Non-invasive detection of urothelial cancer through the analysis of driver gene mutations and aneuploidy. *eLife* **7**:e32143. doi: 10.7554/eLife.32143.

Published 20, March 2018

The affiliation of Luis A Diaz was incorrect. The correct affiliation is: Department of Medicine, Memorial Sloan Kettering Cancer Center, New York, United States. In addition, Luis Diaz would like to declare the following for his competing interests statement:

Luis A Diaz (LAD) is a member of the board of directors of Personal Genome Diagnostics (PGDx) and Jounce Therapeutics. LAD holds equity in PapGene, Personal Genome Diagnostics (PGDx) and Phoremost. He is a paid consultant for Merck, PGDx and Phoremost. LAD is an inventor of licensed intellectual property related to technology for ctDNA analyses and mismatch repair deficiency for diagnosis and therapy from Johns Hopkins University. These licenses and relationships are associated with equity or royalty payments to LAD. The terms of all these arrangements are being managed by Johns Hopkins and Memorial Sloan Kettering in accordance with their conflict of interest policies. In addition, in the past 5 years, LAD has participated as a paid consultant for one-time engagements with Caris, Lyndra, Genocea Biosciences, Illumina and Cell Design Labs.

The article has been corrected accordingly.

